# Decitabine Inhibits Bone Resorption in Periodontitis by Upregulating Anti-Inflammatory Cytokines and Suppressing Osteoclastogenesis

**DOI:** 10.3390/biomedicines9020199

**Published:** 2021-02-17

**Authors:** Urara Tanaka, Shunichi Kajioka, Livia S. Finoti, Daniela B. Palioto, Denis F. Kinane, Manjunatha R. Benakanakere

**Affiliations:** 1 Department of Periodontics, School of Dental Medicine, University of Pennsylvania, Philadelphia, PA 19104, USA; urara@dental.upenn.edu (U.T.); sertorifil@email.chop.edu (L.S.F.); dpalioto@upenn.edu (D.B.P.); 2 Department of Periodontics, Kyushu University Hospital, Maidashi, Higashi-ku, Fukuoka 812-8582, Japan; 3 Department of Pharmaceutical Sciences, School of Pharmacy at Fukuoka, International University of Health Welfare, Fukuoka 812-8582, Japan; kajioka@uro.med.kyushu-u.ac.jp; 4 Center for Applied Genomics, The Children’s Hospital of Philadelphia, PA 19104, USA; 5 Department of OMS and Periodontology, School of Dentistry of Ribeirão Preto, University of São Paulo, Ribeirão Preto 14040-904, Brazil; 6 Periodontology Department, Bern Dental School, University of Bern, 3012 Bern, Switzerland; dfkinane@outlook.com

**Keywords:** periodontitis, periodontal bone-loss, decitabine, Krüppel-like transcription factor-2, interleukin-10, transforming growth factor beta-1

## Abstract

DNA methylation controls several inflammatory genes affecting bone homeostasis. Hitherto, inhibition of DNA methylation in vivo in the context of periodontitis and osteoclastogenesis has not been attempted. Ligature-induced periodontitis in C57BL/6J mice was induced by placing ligature for five days with Decitabine (5-aza-2′-deoxycytidine) (1 mg/kg/day) or vehicle treatment. We evaluated bone resorption, osteoclast differentiation by tartrate-resistant acid phosphatase (TRAP) and mRNA expression of anti-inflammatory molecules using cluster differentiation 14 positive (CD14^+^) monocytes from human peripheral blood. Our data showed that decitabine inhibited bone loss and osteoclast differentiation experimental periodontitis, and suppressed osteoclast CD14^+^ human monocytes; and conversely, that it increased bone mineralization in osteoblastic cell line MC3T3-E1 in a concentration-dependent manner. In addition to increasing *IL10* (interleukin-10), *TGFB* (transforming growth factor beta-1) in CD14^+^ monocytes, decitabine upregulated KLF2 (Krüppel-like factor-2) expression. Overexpression of KLF2 protein enhanced the transcription of *IL10* and *TGFB.* On the contrary, site-directed mutagenesis of *KLF2* binding site in *IL10* and *TFGB* abrogated luciferase activity in HEK293T cells. Decitabine reduces bone loss in a mouse model of periodontitis by inhibiting osteoclastogenesis through the upregulation of anti-inflammatory cytokines via KLF2 dependent mechanisms. DNA methyltransferase inhibitors merit further investigation as a possible novel therapy for periodontitis.

## 1. Introduction

Periodontitis is a chronic inflammatory disease of the gum characterized by the destruction of periodontal tissue by bacteria and, if untreated, progression to heavy alveolar bone loss [1]. The onset and progression of bacterial periodontitis are influenced by various environmental and host-related risk factors [2], and targeting the modifiable risk factors is essential to optimize the response to treatment. The management of periodontitis includes behavioral changes in oral hygiene, subgingival instrumentation, local and system drug therapy, and surgery [3]. Recent years have seen the development of novel regenerative therapies to replace lost periodontal tissue, and some of these treatments have achieved favorable results in a clinical setting [4,5,6]. However, some cases of periodontitis remain intractable despite the application of these new therapies. Therefore, novel treatments for periodontitis are required. Periodontitis is a complex chronic inflammatory disease that causes severe destruction of the tooth structures. Host cell modulators play a pivotal role and directly activate bone eating cells (osteoclasts) to initiate bone loss. In disease state, receptor activator of nuclear factor kappa-Β ligand (RANKL), an activator of osteoclasts, is elevated, whereas osteoclast inhibitor, osteoprotegrin (OPG) is reduced. The ratio of RANKL/OPG is modulated mainly by tumor necrosis factor (TNF) and interleukin-1 (IL-1) released from host cells, subsequently activating osteoclasts, resulting in periodontitis. Hence, osteoclast formation, differentiation and function are important in understanding the disease process. The balance between pro-inflammatory cytokines such as TNF and anti-inflammatory cytokines like IL-10 and TGF-β dictate homeostasis. In addition, genetic components account for majority of control in bone turnover. Due to genetic variation, some individuals exhibit severe alveolar bone loss and high levels of inflammatory cytokines compared to other individuals that cannot be explained by the presence of pathogenic microbe. Recently, we demonstrated that epigenetic variations in the host play an important role in the development of periodontitis [7,8]. DNA methylation processes control several key inflammatory genes affecting alveolar bone homeostasis [7,8]. 

There is now convincing evidence that epigenetic mechanisms such as DNA methylation are involved in the pathogenesis of periodontitis [9]. For example, it has been reported that tissue affected by periodontitis and normal periodontal tissue show differences in the methylation status of promoters for inflammation-related genes such as those encoding interleukin (IL)-8, tumor necrosis factor-alpha (TNFα) and toll-like receptor-2 (TLR2) [10,11,12,13]. Furthermore, increased methylation of the *TLR2* promoter has been reported in a mouse model of periodontitis caused by infection with *Porphyromonas gingivalis* [7]. The above observations raise the possibility that epigenetic therapies could be used in the treatment of periodontitis. 

Decitabine is a DNA methyltransferase inhibitor approved by the United States Food and Drug Administration for the treatment of myelodysplastic syndrome (MDS) [14,15]. MDS is characterized by clonal proliferation of neoplastic hematopoietic stem cells of myeloid origin with recurrent genetic abnormalities. The syndrome is also characterized by ineffective hematopoiesis, concurrent peripheral blood cytopenia with a high risk of developing acute myeloid leukemia (AML) [16]. Mutations in hematopoietic cells are thought to be the driver of the MDS. Most mutations occur at CpG sites suggestive of age related DNA methylation [17]. The most common treatment for MDS include hypomethylating agents such as azacytidine and decitabine [18]. Decitabine is incorporated into DNA where it inhibits DNA methyltransferase activity [19], and the resulting reduction in the methylation of CpG islands in promoter regions leads to downregulated gene expression [20,21]. In case of MDS, treatment with Decitabine yielded encouraging response [22,23]. However, no previous studies have described the effects of DNA methyltransferase inhibitors on periodontitis.

We hypothesized that DNA methyltransferase inhibitors could be used as a novel epigenetic therapy for periodontitis. Therefore, the aim of this study was to investigate the effects of decitabine on periodontitis in vitro and in vivo. In particular, we examined whether decitabine modified the expressions of genes encoding anti-inflammatory molecules such as Krüppel-like factor-2 (KLF2), since these molecules inhibit osteoclast activity and thus periodontitis-associated alveolar bone loss.

## 2. Materials and Methods

### 2.1. Mice and Reagents

Female C57BL/6 mice aged 10 weeks were purchased from the Jackson Laboratory (Bar Harbor, ME, USA). Animals were housed under specific pathogen-free conditions, and food and water were provided ad libitum. The animal experiments were approved by the Institutional Animal Care and Use Committee of the University of Pennsylvania (protocol approval no:805456), and all animal experiments complied with the ARRIVE guidelines (Animal Research: Reporting of In Vivo Experiments). Decitabine (5-aza-2′-deoxycytidine; Sigma-Aldrich, St. Louis, MO, USA) was diluted in 2% carboxymethylcellulose and administered to the mice by oral gavage at a dose of 1 mg/kg/day. Vehicle (2% carboxymethylcellulose) was administered as a control. Mice were divided into four groups: (1) control (no periodontitis and administration of vehicle, *n* = 5); (2) decitabine (no periodontitis and administration of decitabine, *n* = 5); (3) periodontitis + vehicle (ligature-induced periodontitis and administration of vehicle, *n* = 5); and (4) periodontitis + decitabine (ligature-induced periodontitis and administration of decitabine, *n* = 5). 

### 2.2. Ligature-Induced Periodontitis Model 

First, 5-0 silk ligature was tied around the maxillary left second molar to induce bacteria-mediated inflammation and bone loss [24]. The ligature remained in place throughout the experimental period (five days), and decitabine or vehicle was administered once every day (a total of five times) by oral gavage. The mice were euthanized by deep anesthesia (isoflurane) five days after placement of the ligature. 

### 2.3. Analysis of Periodontal Bone Loss

Images of the maxillae were obtained using an SMZ800 microscope (×40 objective) equipped with a Digital Sight DS-U3 camera controller (Nikon, Tokyo, Japan), and the distances from the cementoenamel junction (CEJ) to the alveolar bone crest (ABC) were measured. Bone loss was calculated by subtracting the 6-site total CEJ-ABC distance for the ligated side of each mouse from the 6-site total CEJ-ABC distance for the contralateral unligated side. Negative values indicated bone loss relative to the baseline (unligated control) [24].

### 2.4. Staining for Tartrate-Resistant Acid Phosphatase (TRAP) 

The maxillae were fixed in 4% paraformaldehyde for 24 h and decalcified with EDTA for 14 days at 4 °C. The specimens were embedded in optimal cutting temperature compound and cut into 6-µm sections in a mesio-distal direction using a cryostat at −20 °C (Leica, Wetzlar, Germany). The sections were stained for TRAP (Takara, Tokyo, Japan), and TRAP-positive multinucleated cells (three or more nuclei) were counted in four random sections from each mouse.

### 2.5. Immunohistochemistry

Mesio-distal sections were incubated with rabbit anti-KLF2 polyclonal primary antibody (1:200 dilution; ab203591; Abcam, Cambridge, UK) overnight at 4 °C. The sections were then incubated with secondary antibody (Alexa Fluor 488-conjugated goat anti-rabbit IgG; 1:200 dilution; ab150077; Abcam) and covered with Prolong Gold Reagent with DAPI (Molecular Probes, Thermo Fisher Scientific, Waltham, MA, USA). The sections were visualized using an Eclipse NiE automated upright fluorescence microscope (Nikon).

### 2.6. Osteoclast Differentiation

CD14^+^ monocytes were isolated from human peripheral blood mononuclear cells (PBMCs, obtained from the Human Immunology Core, University of Pennsylvania) using the Human CD14 Positive Selection Kit (STEMCELL Technologies, Vancouver, Canada). After isolation, CD14^+^ cells were cultured in Minimum Essential Medium Alpha (MEMα) containing macrophage colony-stimulating factor (M-CSF; 20 ng/mL; Abcam) for 24 h. The cells were collected, seeded in 96-well plates (5 × 10^4^ cells/well) and cultured in MEMα containing M-CSF (20 ng/mL), receptor activator of nuclear factor kappa-Β ligand (RANKL; 40 ng/mL; R&D Systems, Minneapolis, MN, USA) and decitabine (0, 0.5, 1, 10 or 100 µM) for five days. Then, the cells were stained for TRAP and alkaline phosphatase (ALP) using the TRAPCP&ALP Double-stain Kit (Takara). TRAP-positive multinucleated cells (three or more nuclei) were counted as osteoclast-like cells.

### 2.7. Osteoblastic Cell Line MC3T3-E1 Culture and Osteogenic Differentiation

MC3T3-E1 cells were cultured in osteogenic differentiation medium consisting of MEMα supplemented with 10 nM dexamethasone, 50 μg/mL L-ascorbic acid, 10 mM β-glycerophosphate for adequate periods to perform each experimental protocol (three days for ALP assay, two weeks for osteoblastic mineralization assay). Decitabine was added at each concentration (0, 1, 10 or 100 µM). ALP activities were measured using the ALP Assay Kit^®^ (TAKARA, Shiga, Japan). Bone mineralizattion was measured using OsteoImage Mineralization Assay (Lonza, Walkersville, MD, USA).

### 2.8. Real-Time PCR

CD14^+^ monocytes were cultured in osteoclast differentiation medium with decitabine for three days, and total RNA was isolated from the cells using the RNeasy Kit (Qiagen Silicon Valley, Redwood City, CA, USA). RNA integrity and quantity were checked using a NanoDrop ND1000 spectrophotometer (Thermo Fisher Scientific). cDNA was synthesized from 5 µg total RNA using the High Capacity cDNA Reverse Transcription Kit (Applied Biosystems, Carlsbad, CA, USA). Real-time PCR was performed using an ABI 7500 Fast system (Applied Biosystems) together with TaqMan primers and probe sets (Applied Biosystems) for *IL10* (which encodes IL-10; Hs00961622_m1), *TGFB1* (which encodes transforming growth factor beta-1, *TGFβ1* (Hs00998133_m1) *KLF2* (Hs00360439_g1) and *GAPDH* (which encodes glyceraldehyde 3-phosphate dehydrogenase, *GAPDH* (Hs02786624_g1). *GAPDH* expression was used as an endogenous control, and the relative levels of *IL10*, *TGFβ1* and *KLF2* mRNA were determined using the 2^−ΔCT^ method. 

### 2.9. Luciferase Assay

The KLF2-binding sequences in the promoter regions of *IL10* and *TGFB1* were identified using the Genomatix software suite (Genomatix, Munich, Germany). To construct each promoter reporter, the base pairs were amplified by PCR using the following primers (Thermo Fisher Scientific): *IL10* (−846 to −164): forward, 5′-TAAGCACTCGAGTGCCTCAGTTTGCTCACTATAA-3′, reverse, 5′-TGCTTAAGATCTTAGAGCTCCTCCTTCTCTAACC-3′; *TGFB1* (−5329 to −1936): forward, 5′-TAAGCACTCGAGCACCTTGGTCAGTCTCCTATAAC-3′, reverse, 5′-TGCTTAAGATCTGCCTCTGCCCTAATCATACAA-3′; *TGFB1* (−3063 to −1936): forward, 5′-TAAGCACTCGAGATCACAAGGCTCTCCACAAC-3′, reverse, 5′-TGCTTAAGATCTCATCTCATGCTGATCCCTTCTC-3′; *TGFB1* (−2144 to −1936): forward, 5′-TAAGCACTCGAGGGGATAGATAAGACGGTGGGA-3′, reverse, 5′-TGCTTAAGATCTGGGACCACACCTGGAAATG-3′. The PCR products were subcloned into the pGL4.20[luc2/Puro] vector (Promega, Madison, WI, USA) and transformed into One Shot TOP10 Competent Cells (Invitrogen, Thermo Fisher Scientific). Transformed clones were selected on LB plates with 100 mg/mL ampicillin (Sigma-Aldrich). Similar fragments with the same promoter region sequence of each gene (*IL10* and *TGFβ1*) without KFL2 binding sites were generated using gBlocks^®^ Gene Fragments (Integrated DNA Technologies, Coralville, IA, USA) and cloned in the pGL4.20[luc2/Puro] vector. On the day before transfection, HEK293T cells were plated at a density of 5 × 10^5^ cells per six-well plate and incubated in pre-warmed DMEM supplemented with 10% fetal bovine serum and 1% penicillin/streptomycin at 37 °C and 5% CO_2_. The cells were transfected with either reporter plasmid containing the KLF2 biding site or reporter plasmid not containing the KLF2 biding site (500 ng), and in both cases the cells were simultaneously co-transfected with either PpyCAG-KLF2-IB expression plasmid (100 ng; Addgene, Watertown, MA, USA) or MOCK plasmid (control) using the GenMute Reagent (SignaGen Laboratories, Frederick, MD, USA) in accordance with the manufacturer’s instructions. Luciferase activity was assessed at 24 h after transfection using the Dual-Luciferase Reporter Assay system (E1910; Promega). All transfections were performed in duplicate for at least three independent experiments.

### 2.10. Statistical Analysis

All experiments were repeated at least three times for statistical interpretation. Experimental data are reported as the mean ± standard deviation (SD). Comparisons between groups were made using the paired t-test. A *p*-value < 0.05 was considered to indicate a significant difference. 

## 3. Results

### 3.1. Decitabine Inhibits Bone Resorption in Mice with Ligature-Induced Periodontitis

**Figure 1:**
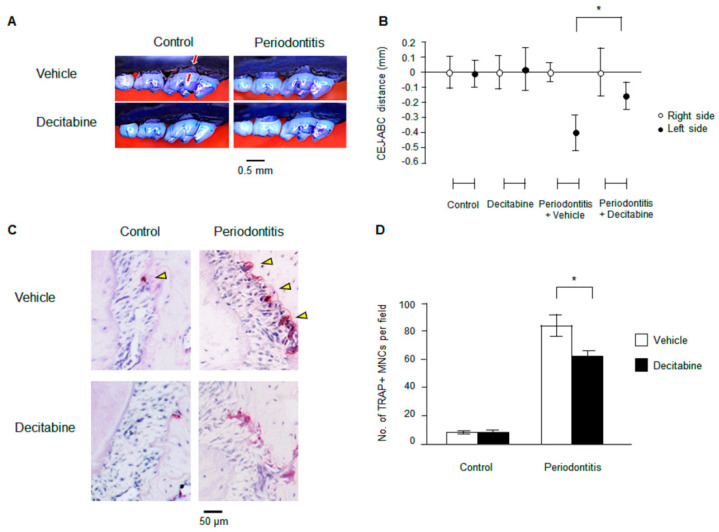
Effects of decitabine on bone resorption and osteoclastogenesis in a mouse model of ligature-induced periodontitis. (**A**) Representative images illustrating bone loss in the mouse model of periodontitis. The red arrows show the distance from the cementoenamel junction to the alveolar bone crest (CEJ-ABC distance), and bone loss is indicated in light purple. (**B**) Averaged data for the CEJ-ABC distance in the four groups. Negative values (mm) indicate bone loss relative to the unligated control side. * *p* < 0.05 vs. periodontitis + vehicle group. (**C**) Representative images showing tartrate-resistant acid phosphatase-positive multinuclear osteoclasts (TRAP+ MNCs; yellow arrows). Magnification: ×40. (**D**) Averaged data for the number of osteoclasts per high-power field. * *p* < 0.05 vs. periodontitis + vehicle group.

Mice with ligature-induced periodontitis exhibited heavy bone loss compared with control mice (Figure 1A). However, the administration of decitabine by oral gavage for five days resulted in a marked reduction in the degree of bone loss in mice with periodontitis (Figure 1A). As summarized in Figure 1B, there was a significant difference in CEJ-ABC distance between mice with ligature-induced periodontitis treated with decitabine and those administered vehicle (−0.15 ± 0.09 mm vs. −0.40 ± 0.12 mm, *n* = 5, *p* < 0.05). The above results indicate that decitabine can protect against bone loss in a mouse model of periodontitis. 

### 3.2. Decitabine Suppresses Osteoclastogenesis in Mice with Ligature-Induced Periodontitis

Osteoclast activity is essential for the alveolar bone resorption that occurs in periodontitis. Therefore, we assessed whether decitabine might regulate osteoclast differentiation. Staining for TRAP revealed that periodontitis significantly increased the number of osteoclasts in alveolar bone (Figure 1C,D). Furthermore, mice with periodontitis treated with decitabine had significantly fewer osteoclasts in alveolar bone than mice with periodontitis administered vehicle (57.4 ± 6.12 vs. 79.6 ± 8.02 per high-power field, *n* = 5, *p* < 0.05; Figure 1C,D). 

### 3.3. Decitabine Decreases Osteoclastogenesis in Human CD14^+^ Monocytes In Vitro in a Dose-Dependent Manner

**Figure 2:**
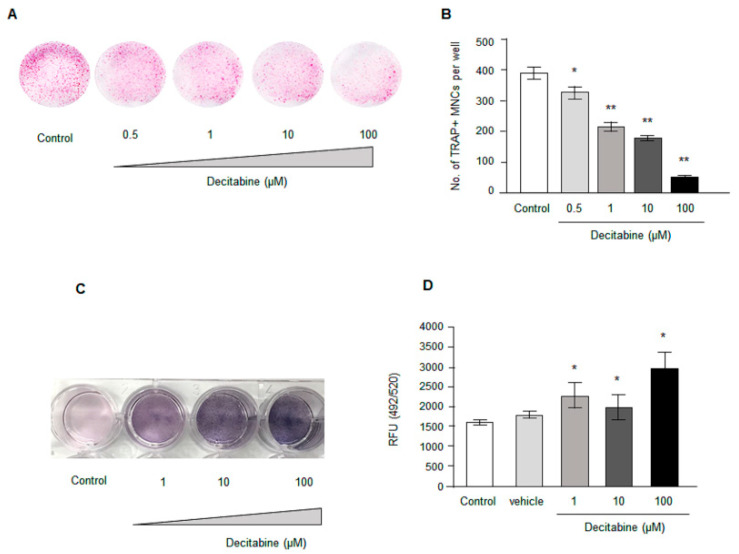
Effects of decitabine on osteoclast differentiation in vitro. (**A**) Representative images showing osteoclastogenesis in CD14^+^ monocytes in the presence of various concentrations of decitabine (0.1, 1, 10 or 100 µM). (**B**) Averaged data for the number of tartrate-resistant acid phosphatase-positive multinuclear osteoclasts (TRAP+ MNCs) per well. (**C**) MC3T3-E1 cells cultured in osteogenic differentiation media in the presence of Decitabine for three days. ALP staining was performed after osteogenic stimulation. (**D**) MC3T3-E1 cells cultured in osteogenic differentiation media in the presence of Decitabine for two weeks. Decitabine dose dependently increased osteo-mineralization. * *p* < 0.05, ** *p* < 0.01 vs. control. Note that vehicle indicates the osteogenic differentiation media with 0.1% DMSO.

To determine whether the effects of decitabine on osteoclast numbers in vivo might be due to inhibition of osteoclastogenesis, we next examined whether decitabine (0.5–100 µM) influenced osteoclast differentiation in vitro using CD14^+^ monocytes cultured in M-CSF and RANKL. Decitabine was found to suppress osteoclast differentiation in a concentration-dependent manner (Figure 2A,B): the number of TRAP-positive cells was significantly lower for 0.5 µM decitabine (325.25 ± 18.7, *n* = 4, *p* < 0.05), 1 µM decitabine (216.0 ± 13.2, *n* = 4, *p* < 0.01), 10 µM decitabine (181.8 ± 5.5, *n* = 4, *p* < 0.01) and 100 µM decitabine (56.5 ± 4.7, *n* = 4, *p* < 0.01) than for the control (387.8 ± 16.6, *n* = 4).

### 3.4. Decitabine Increases Osteogenic Differentiation in Osteoblast Precursor Cell Line MC3CT3-E1 in a Dose Dependent Manner

The observation that Decitabine inhibited osteoclast formation and resisted alveolar bone resorption in vivo prompted us to test whether Decitabine influenced osteogenic differentiation. We utilized the MC3T3-E1 cell line to test the effect of osteogenic differentiation in the presence of Decitabine at different concentrations. An osteoblastic mineralization assay and ALP assay were performed to determine osteogenic property. Interestingly, Decitabine dose-dependently increased osteogenic differentiation in MC3T3-E1 cells (Figure 2C,D). Taken together our data suggest that DNA demethylation by Decitabine not only inhibited osteoclastogenesis but also promoted osteogenic differentiation.

### 3.5. Decitabine Increases the Expression of Anti-Inflammatory Cytokines in CD14^+^ Monocytes

**Figure 3:**
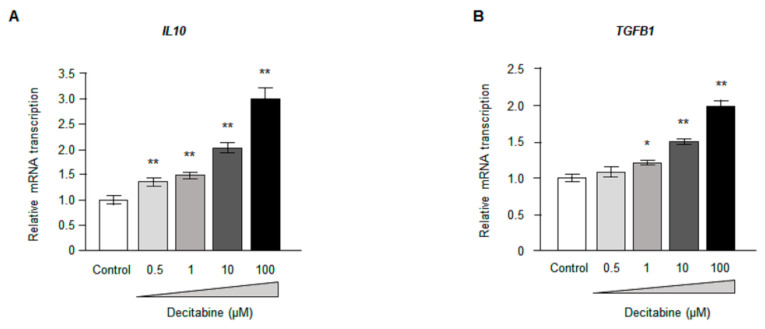
Decitabine dose dependently increased anti-inflammatory cytokine in vitro. (**A**,**B**) mRNA expression of *IL10* (**A**) and *TGFB1* (**B**) measured by real-time PCR. The mRNA level was normalized to the mean control value. * *p* < 0.05, ** *p* < 0.01 vs. control.

In addition to inhibiting osteoclastogenesis (see above), decitabine also caused a dose-dependent increase in the mRNA expression of two important anti-inflammatory cytokines in vitro (Figure 3A,B). The relative expression of *IL10* (Figure 3A) was significantly higher for 0.5 μM decitabine (1.38 ± 0.06, *n* = 4, *p* < 0.01), 1 μM decitabine (1.49 ± 0.07, *n* = 4, *p* < 0.01), 10 μM decitabine (2.02 ± 0.09, *n* = 4, *p* < 0.01) and 100 μM decitabine (2.98 ± 0.25, *n* = 4, *p* < 0.01) than for the control (1.01 ± 0.08, *n* = 4). Furthermore, the relative expression of *TGFβ1* (Figure 3B) was significantly higher for 1 μM decitabine (1.22 ± 0.02, *n* = 4, *p* < 0.05), 10 μM decitabine (1.50 ± 0.04, *n* = 4, *p* < 0.01) and 100 μM decitabine (1.98 ± 0.10, *n* = 4, *p* < 0.01) than for the control (1.00 ± 0.05, *n* = 4).

### 3.6. Decitabine Increases the Expression of KLF2

**Figure 4:**
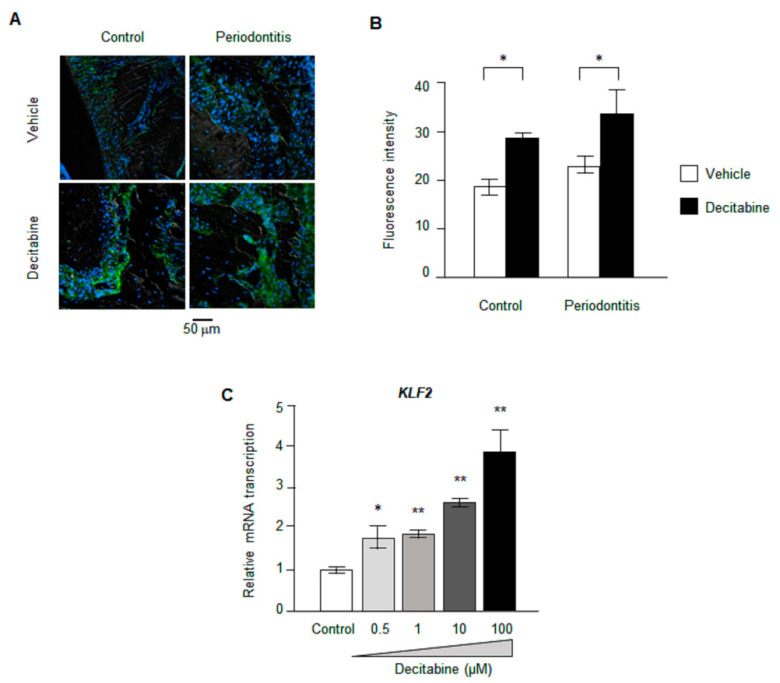
Effects of decitabine on Krüppel-like factor-2 (KLF2) expression in vitro and in vivo. (**A**) Representative immunofluorescence images showing the expression of KLF2 protein. The tissues were immunostained for KLF2 (green), and nuclei were counterstained with 4′,6-diamidino-2-phenylindole (DAPI) (blue). Magnification: ×200. (**B**) Averaged data for the fluorescence intensity of KLF2 immunostaining (i.e., KLF2 protein expression) in each group. * *p* < 0.05 vs. corresponding vehicle group. (**C**) *KLF2* mRNA expression in vitro measured by real-time PCR. The mRNA level was normalized to the mean control value. * *p* < 0.05, ** *p* < 0.01 vs. control.

It is widely recognized that KLF2 exerts an anti-inflammatory effect by regulating the expression of various cytokines. Since *KLF2* expression is altered by methylation of its promoter (Yan et al., 2017) [25], we considered KLF2 to be a potential candidate mediating the effects of decitabine on anti-inflammatory cytokines. Immunohistochemistry experiments revealed no significant difference in KLF2 protein expression between mice with ligature-induced periodontitis and control animals (Figure 3A,B). However, decitabine significantly increased KLF2 protein expression in mice with periodontitis as well as in control mice (Figure 4A,B). Furthermore, real-time PCR demonstrated that decitabine caused a concentration-dependent enhancement of *KLF2* mRNA expression in CD14^+^ monocytes cultured in vitro (Figure 4C): relative *KLF2* mRNA expression was significantly higher for 0.5 μM decitabine (1.81 ± 0.27, *n* = 4, *p* < 0.05), 1 μM decitabine (1.88 ± 0.10, *n* = 4, *p* < 0.01), 10 μM decitabine (2.65 ± 0.11, *n* = 4, *p* < 0.01) and 100 μM decitabine (3.88 ± 0.55, *n* = 4, *p* < 0.01) than for the control (1.01 ± 0.07, *n* = 4). 

### 3.7. KLF2 Upregulates the Transcription of Genes Encoding Anti-Inflammatory Cytokines 

**Figure 5:**
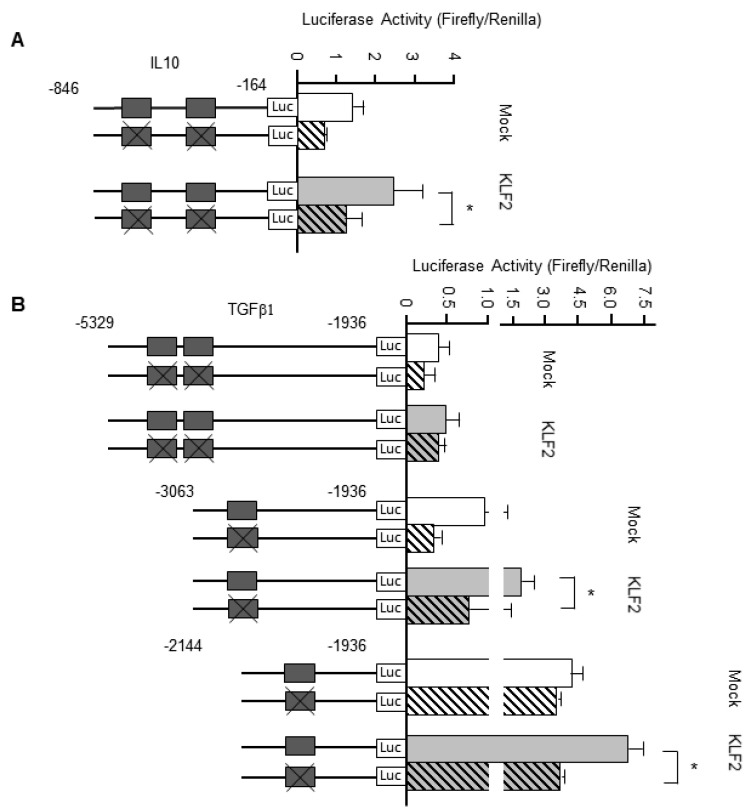
Effects of Krüppel-like factor-2 (KLF2) overexpression on genes encoding anti-inflammatory cytokines. HEK293T cells were co-transfected with the indicated luciferase reporter for interleukin (IL)-10 or transforming growth factor beta-1 (TGFβ1) expression and either a KLF2 overexpression plasmid (KLF2 columns) or empty vector (mock columns). Luciferase activity was normalized to *Renilla* luciferase activity. (**A**) Averaged data for luciferase activity in experiments using the luciferase reporter for IL-10. * *p* < 0.05 vs. IL-10 promoter with KLF2 overexpression plasmid. (**B**) Averaged data for luciferase activity in experiments using the luciferase reporter for TGFβ1. * *p* < 0.05 vs. TGFβ1 promoter with KLF2 overexpression plasmid.

Additional in vitro experiments were carried out to establish whether KLF2 could regulate the transcription of genes encoding two anti-inflammatory cytokines, namely IL-10 and TGFβ1. HEK293T cells transfected with a KLF2 expression plasmid and luciferase vector were cultured for 24 h, following which the luciferase activity was measured. In experiments monitoring *IL10* gene transcription (Figure 5A), overexpression of KLF2 resulted in a significantly higher luciferase activity when KLF2 binding sites were present in the promoter than when KLF2 binding sites were absent (2.44 ± 0.73 vs. 1.22 ± 0.41, *n* = 4, *p* < 0.05). Similar results were obtained when *TGFB1* gene expression was examined (Figure 5B): luciferase activity was higher when KLF2 binding sites were present in the promoter than when KLF2 binding sites were absent (1.90 ± 0.57 vs. 0.75 ± 0.68, *n* = 4, *p* < 0.05). 

## 4. Discussion

**Figure 6:**
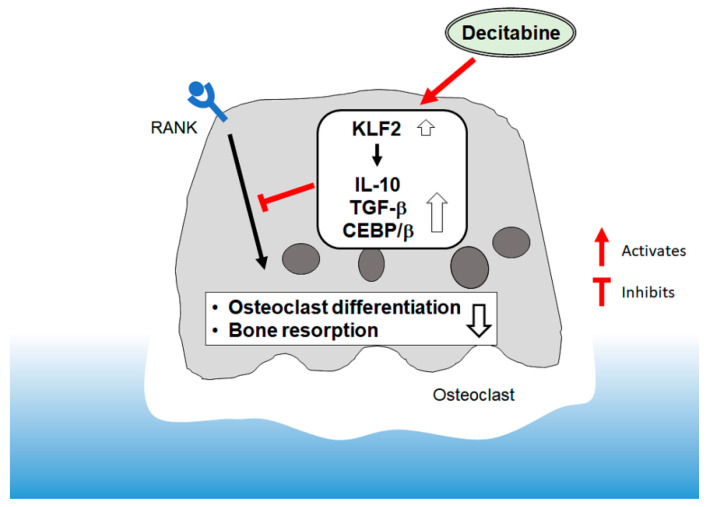
Schematic figure illustrating the possible mechanisms underlying the inhibitory action of decitabine on osteoclast differentiation.

The present study found that decitabine suppressed osteoclast differentiation in a concentration-dependent manner. Despite inhibiting osteoclast differentiation, decitabine activated CD14^+^ monocytes to increase their expression of *IL10* and *TGFβ1* mRNA. Furthermore, the expression of KLF2 was increased at both the gene and protein level, and overexpression of KLF2 enhanced the transcription of *IL10* and *TGFβ1* in HEK293T cells. Taken together, our findings suggest that decitabine inhibits bone resorption in mice with periodontitis by upregulating *KLF2* expression and thereby enhancing the expression of *IL10* and *TGFβ1* (Figure 6).

Recently it has been reported that epigenetic mechanisms, including DNA methylation, regulate genes related to host immune responses to periodontal pathogens [26,27,28]. This raises the possibility that epigenetic modifications in periodontal tissue could be novel therapeutic targets for the treatment of periodontitis. However, there are limited published data on the effects of DNA methyltransferase inhibitors on periodontitis. Since decitabine is already in clinical use for the treatment of myelodysplastic syndromes, we evaluated the effects of this DNA methyltransferase inhibitor on periodontitis in vivo and on osteoclast differentiation and gene expression in vitro. Osteoclast activity is essential for periodontitis-associated bone resorption. We found that decitabine inhibited bone resorption in a mouse model of periodontitis, reduced the number of osteoclasts and suppressed osteoclastogenesis. Our observation that decitabine suppressed the osteoclastic differentiation of CD14^+^ monocytes is consistent with a previous report that decitabine inhibited the osteoclastic differentiation of RAW264.7 cells, which are a monocyte/macrophage-like cell line [29]. It is well known that RANK-RANKL signaling is indispensable for the activation of osteoclastogenesis, and this signaling pathway is regulated by inflammatory cytokines [30,31]. Therefore, we focused on the effects of decitabine on two representative anti-inflammatory cytokines, IL-10 and TGFβ1. We found that decitabine increased the mRNA expression of *IL10* and *TGFβ1* in osteoclasts. IL-10 is an anti-inflammatory cytokine that inhibits the synthesis of pro-inflammatory cytokines and suppresses osteoclast differentiation [32,33,34]. TGFβ1 regulates immune cells and tissue repair processes and suppresses osteoclast differentiation in peripheral blood mononuclear cells (PBMCs) [35]. Taken together, our results raise the possibility that decitabine may show promise as a new treatment for periodontitis. Moreover, the proof that decitabine inhibited bone loss by osteoclast suppression and osteoblast differentiation in experimental periodontitis, this could be extended to other bone diseases associated with periodontitis, such as osteonecrosis and/or osteoporosis and rheumatoid arthritis. Interestingly, osteoporosis and periodontal disease share bidirectional relationship due to closely associated risk factors such as age, lifestyle habits, genetic factors, systemic disease, menopause in women, and dysbiotic oral and gut microbiota [36]. Patients with osteoporosis exhibit loss of bone mineral density along with bone loss at the level of alveolar bone. This newly resorbed alveolar bone could form deeper periodontal pockets harboring pathogenic microbes and instigating proinflammatory cytokines, epigenetic alterations, and increased osteoclast activity resulting in severe periodontal disease [36]. This bidirectional association also extends periodontal disease in the onset of rheumatoid arthritis due to systemic inflammatory complications mediated by periodontopathogens [37].

KLF2 is recognized as an important regulator of inflammation [38,39], so our subsequent experiments focused on the relationships between KLF2 and inflammatory cytokines. Since KLF2 has CpG islands in the promoter region where DNA methylation occurs [25], we speculated that demethylation of KLF2 by decitabine might be a promising method of modifying cytokine levels and thereby reducing the resorption of periodontal bone by osteoclasts. In this study, we showed that decitabine increased *KLF2* mRNA expression in vitro and KLF2 protein expression in vivo. Moreover, experiments in HEK293T cells transfected with an IL-10 or TGFB1 reporter plasmid indicated that the overexpression of KLF2 enhanced the transcription of the *IL10* and *TGFB1* genes. We propose that decitabine increases the expression of KLF2, which promotes the mRNA expression of *IL10* and *TGFB1* and thereby inhibits the differentiation of osteoclasts. KLF2 is a master regulator not only inhibits osteoclasts but also participates in the differentiation of osteoblasts in bone remodeling [40]. In agreement with this finding, our data with decitabine treatment in MC3CT-E1 cells showed dose dependent increase in osteogenic differentiation coupled with KLF2 gene expression. Interestingly, the previous study demonstrated that KLF2 mRNA and protein expression in MC3CT-E1 cells increased with time in the presence of osteogenic media. The underlying mechanism has been clarified that KLF2 were directly interacted with *Runx2* to induce osteogenic differentiation [40]. Our data concur with the above observation whereby decitabine increased KLF2 expression and modulated osteoclast inhibition as well as promoted osteogenic differentiation.

In this study, decitabine was administrated by oral gavage, which increases the risk of adverse effects due to off-target actions on gene expression in other organs or tissues. Local administration of decitabine, such as by gargling or subgingival administration, might allow higher concentrations of decitabine to be used while minimizing systemic adverse effects. In humans, a randomized clinical trial involving 116 patients (84 adults with MDS), 20 mg of decitabine was administered per square meter of body surface area per day for 10 consecutive days. Major systemic adverse events included mild to moderate neutropenia and thrombocytopenia or other infectious events [41]. Despite these side effects, decitabine is commonly used as a single agent therapy in MDS. The dose of decitabine used for the treatment of myelodysplastic syndrome is 20 mg/m^2^ [42], and it is generally estimated that the dose applied to a mouse is one-fifteenth that administered to a human, so the dose of decitabine used in this study was acceptable. Moreover, the predicted blood concentration in this study would be in the order of micromolar. Notably, we confirmed significant increases in the expressions of KLF2, IL-10 and TGFβ1 in vitro at decitabine concentrations of 0.5 µM and 1 µM, which are pharmacologically permissible doses.

## 5. Conclusions

We demonstrated that the DNA methyltransferase inhibitor, decitabine, ameliorates bone loss in a mouse model of periodontitis via a mechanism that involves the regulation of anti-inflammatory cytokine expression by KLF2. Additional research is needed to evaluate the effects of decitabine on the DNA methylation status of periodontal tissue, especially in the KLF2 promoter region. Nevertheless, our results provide a new insight into anti-inflammatory cytokine regulation via KLF2 and the possible therapeutic role of DNA methyltransferase inhibitors in the treatment of periodontitis.

## Data Availability

The data that support the findings of this study are available from the corresponding authors upon reasonable request.
